# Relationship Between Social Support and Global Cognition by Level of Neighborhood Disadvantage in the HABS‐HD Cohort

**DOI:** 10.1002/alz.086841

**Published:** 2025-01-09

**Authors:** Tasha Rhoads, Kimberly Cobos, Sid E. O'Bryant, Justin B Miller, the HABS‐HD Study Team

**Affiliations:** ^1^ Cleveland Clinic, Cleveland, OH USA; ^2^ Cleveland Clinic Lou Ruvo Center for Brain Health, Las Vegas, NV USA; ^3^ University of North Texas Health Science Center, Fort Worth, TX USA

## Abstract

**Background:**

Prior research has demonstrated the positive association between social support and cognition. Specifically, greater social support has been linked with improved cognitive performance and reduced risk of dementia. In particular, emotional support has been identified as a key dimension in the relationship between social support and cognition. This study examined how the relationship between social support and cognitive functioning varies by neighborhood disadvantage, as measured by the Area Deprivation Index (ADI), in a diverse sample of older adults.

**Method:**

Data from 2811 participants (629 Non‐Hispanic Black, 1107 Hispanic, 1075 non‐Hispanic White individuals) from the community‐based Health & Aging Brain Study–Health Disparities (HABS‐HD) cohort were examined. Three composite social support subscales were computed based on self‐report questionnaire responses to describe the following dimensions: Companion support (e.g., accompanying an individual during activities), Emotional support (e.g., having others to confide in or turn to for advice), and Assistance support (e.g., providing help with household tasks). Separate regressions were stratified by ADI quintile. Each regression included all subscales as predictors along with age, sex, and education as covariates. The Mini‐Mental State Exam (MMSE) Total Score was used to reflect global cognition (outcome).

**Result:**

Regressions indicated overall significant models for each level of neighborhood disadvantage. Within ADI 1 (least disadvantage), ADI 2, and ADI 5 (greatest disadvantage), Assistance support emerged as a significant predictor of global cognition. Within ADI 2, Companion support also significantly predicted global cognition. Finally, within ADI 3, Emotional support significantly predicted global cognition. There were no significant social support subscale predictors evident within ADI 4.

**Conclusion:**

Overall, these findings highlight the generally robust, protective effect of social support on cognitive abilities in this large, diverse cohort of older adults. However, variations across subtypes of social support between neighborhood contexts were evident. This highlights the importance of social support for brain health among individuals across levels of socioeconomic status, although these effects may be somewhat weaker at higher levels of neighborhood disadvantage. This finding underscores the fact that greater resources to promote brain health should be directed towards individuals living in areas of greater neighborhood disadvantage.

